# Theoretical Insights into Ferromagnetism in Sm-Doped CeO_2_ from Hybrid Functional: Synergistic Interplay between Sm and Oxygen Vacancy

**DOI:** 10.1021/acsomega.6c04304

**Published:** 2026-08-21

**Authors:** Xiaoping Han, Maamar Benkraouda, Shahida Maqsood, Deepa Jithin, El Hadi Sadki, Noureddine Amrane

**Affiliations:** Department of Physics, 11239 United Arab Emirates University, P. O. Box Al-Ain 15551, U.A.E

## Abstract

Sm-doped CeO_2_ has attracted considerable attention due to its reported room-temperature ferromagnetism and potential applications in oxide-based spintronic devices. However, the microscopic origin of Sm-induced magnetism and its correlation with oxygen vacancy (*V*_O_) remain elusive. In this work, the hybrid functional method is employed to systematically investigate the electronic structure, defect energetics, and magnetic properties of Sm-doped CeO_2_. Results show that the Sm dopant cannot introduce spin polarization, instead strongly promotes the *V*_O_ formation and preferentially forms a stable Sm–*V*_O_ complex. The formation of such complex stabilizes the singly charged vacancy (*V*_*O*_^+^), which induces a Ce^3+^ center via localization of an unpaired electron on a neighboring Ce ion, resulting in a bound magnetic polaron (BMP). The polaron radius of BMP and the corresponding critical concentration required for BMP overlap and percolation are quantitatively evaluated, providing key insight into the conditions necessary for long-range ferromagnetic ordering. The combined first-principles calculations and percolation analysis support a physically consistent interpretation in which the experimentally observed ferromagnetism can be rationalized by BMP-mediated interactions associated with singly charged oxygen vacancies, arising from the synergistic interplay between Sm dopants and oxygen vacancies. This work offers practical design guidelines for engineering CeO_2_-based dilute magnetic oxides for spintronic applications.

## Graphical abstract

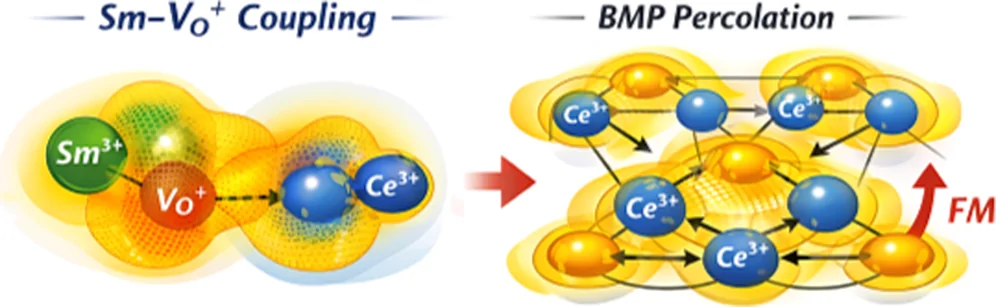

## 1 Introduction

Room-temperature ferromagnetism (RTFM) in wide-bandgap oxide semiconductors has emerged as a central topic in spintronics, offering a promising route to integrate magnetic functionality into conventional semiconductor platforms. Among the various candidates, CeO_2_ has attracted particular attention due to its fluorite crystal structure, high oxygen-ion mobility, and the coexistence of Ce^4+^/Ce^3+^ redox chemistry. This unique electronic flexibility enables strong coupling among charge, defect, and magnetic degrees of freedom, making CeO_2_ an ideal platform for exploring defect-mediated magnetism. Extensive experimental studies have demonstrated that ferromagnetism can be induced in CeO_2_ through doping with transition metals (e.g., Co,^1,2^ Fe,^3^ Cr^4^) or rare-earth elements (e.g., Gd,^5,6^ Ho,^7^ Eu,^8^ Dy,^9^ Pr,^10–12^ Sm^13–17^ ). Among these dopants, Sm has become one of the most intensively studied systems. A variety of synthesis routes—including coprecipitation,^13^ chemical precipitation,^14^ hydrothermal methods,^15^ and polymer pyrolysis^16,17^ —have consistently reported robust RTFM in Sm-doped CeO_2_, suggesting that Sm incorporation is an effective strategy for activating magnetic ordering in this material.

Despite these advances, the microscopic origin of ferromagnetism in Sm-doped CeO_2_ remains highly controversial. Several studies attributed the observed magnetism to the F-center exchange (FCE) mechanism, in which electrons trapped at oxygen vacancies mediate magnetic coupling between localized moments.^13,14,17^ In contrast, alternative interpretations based on double–exchange interactions have been proposed, involving charge transfer and orbital overlap along Sm–O–Sm pathways.^15^ Moreover, Swatsitang et al.^16^ suggested that ferromagnetism originates from oxygen-vacancy-associated bound magnetic polarons (BMPs), whose spatial overlap leads to long-range magnetic ordering. The coexistence of these competing models highlights the lack of a unified and quantitative understanding of Sm-induced ferromagnetism in CeO_2_ and calls for a more rigorous microscopic investigation.

Notably, regardless of the proposed mechanism, oxygen vacancy (*V*_O_) is inevitably involved and plays an indispensable role. As intrinsic defects in CeO_2_, oxygen vacancies introduce excess electrons that preferentially localize on neighboring Ce ions, forming Ce^3+^ states and introducing defect levels within the band gap. Importantly, oxygen vacancies can exist in multiple charge states (neutral, singly charged, and doubly charged), whose relative stability depends sensitively on the Fermi level and chemical environment (oxygen-rich versus oxygen-poor conditions). These features introduce additional complexity but also provide opportunities for tuning magnetic properties via defect engineering. In this context, several critical questions arise: (i) what is the energetically preferred spatial configuration between Sm and *V*_O_? (ii) how does Sm incorporation modify the formation energy and the charge-state stability of oxygen vacancies? (iii) can dopant–defect interactions selectively stabilize specific vacancy charge states, particularly the magnetically active ones? and (iv) what is the dominant microscopic exchange mechanism governing magnetic ordering? Addressing these questions is essential for establishing a physically consistent and predictive picture of magnetism in this system.

From a theoretical standpoint, first-principles calculations based on density functional theory (DFT) have been widely employed to investigate defect physics in CeO_2_. However, conventional DFT approaches (e.g., GGA or LDA) fail to accurately describe the strongly localized Ce 4f electrons,^18^ leading to underestimated band gaps and artificial delocalization of defect states. The DFT + U method (DFT with correction of the Hubbard U potential) partially alleviates this issue by introducing on-site Coulomb interactions,^19–21^ but its predictive capability depends sensitively on the choice of Hubbard U parameters, which can result in inconsistencies in describing charge localization and defect energetics.^22,23^ In contrast, hybrid functional methods,^24,25^ which incorporate a fraction of exact exchange, offer a more reliable and parameter-free treatment of localized 4f states, defect levels, and spin polarization. These methods have been demonstrated to significantly improve the accuracy of vacancy formation energies and electronic structures in ceria-based systems.^22,26–28^ Nevertheless, systematic hybrid functional studies explicitly addressing the interplay between Sm dopants and oxygen vacancies—particularly in relation to vacancy charge-state stability and magnetic exchange mechanisms—remain scarce.

Motivated by these considerations, the present work employs the hybrid functional method to systematically investigate the electronic structure, defect energetics, and magnetic properties of Sm-doped CeO_2_. Particular emphasis is placed on the formation and stability of Sm–*V*_O_ complex, the charge-state-dependent behavior of oxygen vacancy, and their role in mediating magnetic interactions. Our results reveal that isolated Sm is intrinsically nonmagnetic but plays a crucial role in promoting oxygen vacancy formation and stabilizing the singly charged vacancy through forming an energetically favorable defect complex. This stabilization leads to the formation of bound magnetic polarons localized on neighboring Ce ions, which act as the fundamental magnetic units. These findings provide theoretical evidence supporting a physically consistent interpretation of the defect-mediated origin of ferromagnetism, linking dopant–defect interactions, charge-state stability, and polaron-mediated exchange within a unified microscopic framework.

## 2 Models and Method

Pristine CeO_2_ crystallizes in a cubic fluorite structure with the space group of *Fm3m*, containing four Ce atoms and eight O atoms per unit cell, as illustrated in Figure 1a. To minimize artificial dopant–dopant interactions arising from periodic boundary conditions, a 2 × 2 × 2 supercell with eight unit cells (Ce_32_O_64_) was employed to construct the Sm-doped structures. The investigation was first performed by substituting one Ce atom with one Sm atom in the supercell, and subsequently one O vacancy was introduced into the Sm-doped supercell to investigate the coupled effects of dopants and intrinsic defects. Their molecular compositions are SmCe_31_O_64_ and SmCe_31_O_63_, corresponding to Sm_*x*_Ce_1‑*x*_O_2_ and Sm_*x*_Ce_1‑*x*_O_2‑*x*_ (*x* = 0.031), respectively. Figure 1b presents a 2 × 2 × 2 CeO_2_ supercell containing a substitutional Sm dopant.

**Figure 1:**
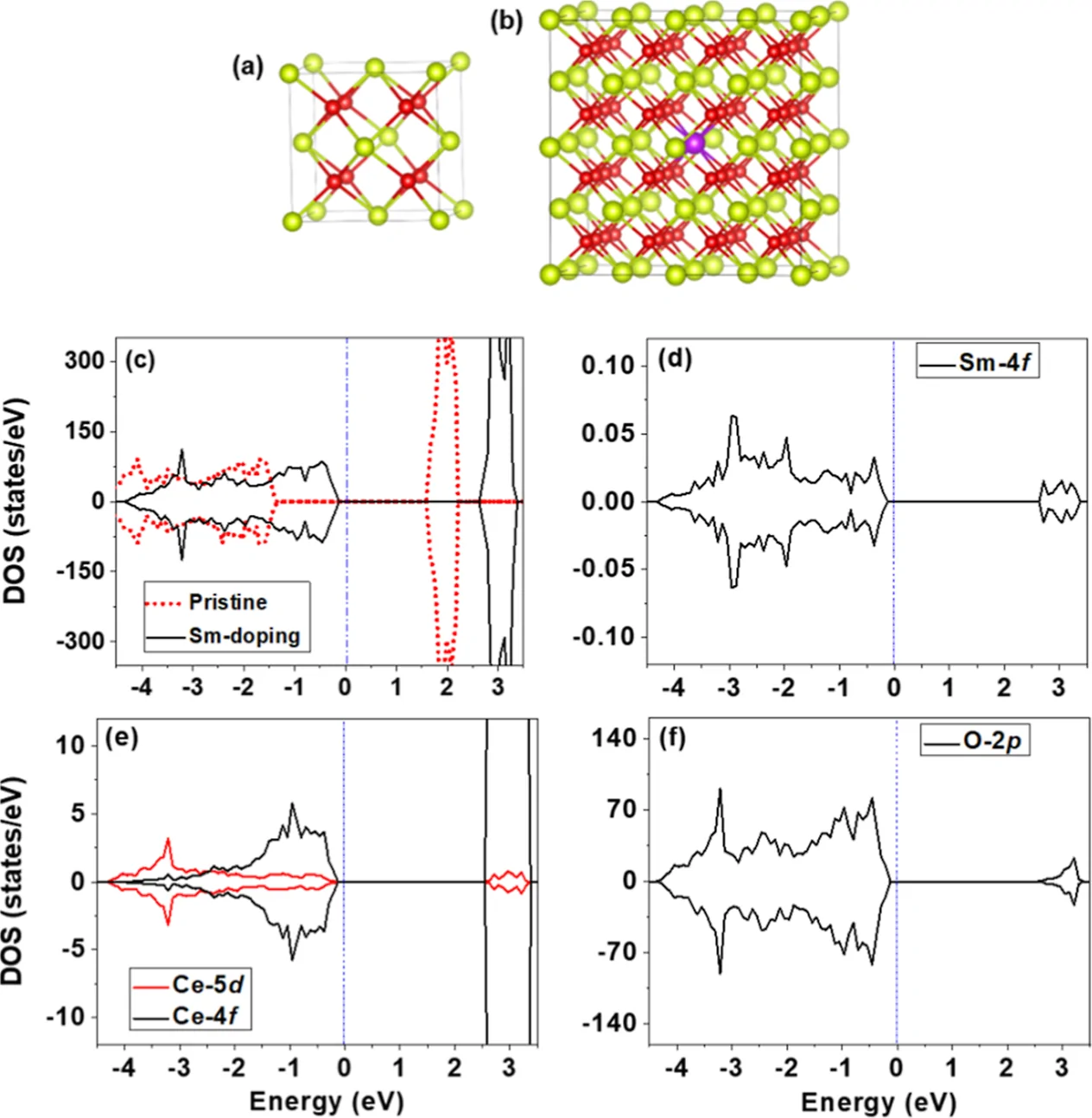
(Color online) (a) CeO_2_ unit cell and (b) 2 × 2 × 2 CeO_2_ supercell with a Sm substitution for Ce. The Ce, O and Sm atoms are represented by green, red and violet spheres, respectively. (c) Calculated DOS for 2 × 2 × 2 CeO_2_ supercell with a Sm substitution for Ce. For the sake of comparison, the DOS of pristine structure is also shown together in red dotted line. The partial DOSs for (d) Sm, (e) Ce and (f) O, respectively.

All theoretical calculations were performed within the framework of spin-polarized DFT as implemented in the Vienna ab initio Simulation Package (VASP).^29,30^ The interaction between ion cores and valence electrons was described using projector augmented-wave (PAW) method.^31^ To accurately capture the strongly localized nature of the Ce 4f states and the defect-induced electronic structure, the Heyd-Scuseria-Ernzerhof (HSE) hybrid functional was employed,^24,25^ including a mixing parameter of 25% Hartree–Fock exchange and a screening parameter of 0.2 Å^–1^. Such hybrid functional with a 25% exact-exchange fraction was adopted throughout this work because it accurately reproduces the experimentally measured structural and electronic properties of CeO_2_. Although modest variations in the mixing parameter may slightly alter the absolute band gap and defect transition energies, previous benchmark studies^27,32–34^ have demonstrated that the localization of Ce 4f electrons, the relative stability of oxygen-vacancy configurations, and the associated defect physics remain qualitatively unchanged. Therefore, the physical conclusions presented in this study are expected to be robust with respect to reasonable variations of the HSE mixing parameter. A plane-wave cutoff energy of 500 eV was used to ensure convergence of total energies and forces. The Brillouin zone was sampled using a Monkhorst–Pack *k*-point grid of 3 × 3 × 3 for the 2 × 2 × 2 supercell.^35^ Electronic self-consistency was achieved with a convergence threshold of 10^–6^ eV, and all atomic positions were fully relaxed until the residual forces on each atom were below 0.01 eV Å^–1^. Spin polarization was included in all calculations to properly account for possible magnetic states associated with defect-induced localized electrons. To validate the reliability of the computational setup, pristine CeO_2_ was first examined. The calculated equilibrium lattice constant (*a* = 5.40 Å) is in excellent agreement with experimental value of 5.41 Å.^36,37^ The calculated band gaps, including the O 2p → Ce 4f gap (3.07 eV) and O 2p → Ce 5d gap (5.96 eV), are also in close agreement with experimental measurements (∼3.0 eV and ∼6.0 eV, respectively).^36,37^ These results confirm that the hybrid functional approach effectively overcomes the well-known band gap underestimation and self-interaction errors inherent in semilocal DFT methods.

## 3 Results & Discussion

First, a system containing a single Sm dopant was investigated, where one Sm atom substitutes a Ce atom in a 2 × 2 × 2 CeO_2_ supercell (see Figure 1b), corresponding to a doping concentration of 3.13%. Its calculated density of states (DOS), shown in Figure 1c, exhibits perfect symmetry between the spin-up and spin-down channels. No discernible spin splitting or asymmetry is observed near the Fermi level or within the band gap, indicating that the system remains nonmagnetic. To further assess the impact of Sm incorporation on electronic structure, the DOSs of the systems with and without Sm doping are compared (see Figure 1c), and it is found that the incorporation of Sm leads to a downward shift of the Fermi level toward the valence band without altering the fundamental band structure. The rigid-band-like behavior confirms that Sm doping primarily introduces p-type character rather than modifying the intrinsic electronic framework. Also, the analyses of the partial DOSs (PDOSs) of Sm (Figure 1d), Ce (Figure 1e) and O (Figure 1f) show that Sm contributes little to the band edges, and that the band-edge characteristics remain essentially unchanged upon Sm substitution: the CBM is still dominated by localized Ce 4f states, while the VBM retains its O 2p character, consistent with pristine CeO_2_. To further clarify the oxidation state of Sm, the electronic population around the Sm site was evaluated by integrating the total charge density within a sphere centered at the Sm atom using its effective ionic radius (1.079 Å).^38^ The result shows that the Sm atom loses approximately 2.68 electrons relative to its neutral state, indicating a stable +3 oxidation state (Sm^3+^). Evidently, the substitution of Ce^4+^ by Sm^3+^ introduces a local charge imbalance, which is accommodated through electronic redistribution rather than spin polarization. Consequently, no localized carriers or spin-polarized states are formed, and the ground state remains nonmagnetic. This finding clearly demonstrates that Sm doping alone is insufficient to induce magnetism in CeO_2_, highlighting the necessity of additional defects to activate magnetic behavior and providing a possible explanation for discrepancies with experimental observations.^13–15^

Considering that oxygen vacancy is intrinsically present in CeO_2_ and can effectively compensate the charge imbalance induced by Sm substitution, an oxygen vacancy was introduced into the above Sm-doped 2 × 2 × 2 CeO_2_ supercell by removing one oxygen atom. To systematically investigate the spatial correlation between Sm and *V*_O_, four configurations were constructed in which *V*_O_ is located at the first (1NN), second (2NN), third (3NN) and fourth nearest-neighboring (4NN) positions relative to the Sm atom, as illustrated in Figure 2a. After full structural relaxation, the 2NN configuration is found to be energetically the most favorable. To quantify the interaction between Sm and *V*_O_, the interaction energy (*E*_int_) was calculated using the following expression^39^

1 $$E_{\mathrm{int}}=E\left(pure:{\;Sm+V}_{O}\right)-E\left(pure:\;Sm\right)-E\left(pure:\;V_{O}\right)+E\left(pure\right)$$

where *E*(pure) is the total energy of the pristine supercell, and *E*(pure: Sm + *V*_O_), *E*(pure: Sm) and *E*(pure: *V*_O_) are the total energies of the supercells containing the Sm-*V*_O_ complex, isolated Sm substitution, and isolated oxygen vacancy, respectively. As summarized in Figure 2b, a stronger attractive interaction is observed in the 2NN configuration (−288 meV), whereas the interaction is much weaker for 1NN (−39 meV) and nearly negligible for 3NN and 4NN. These results indicate a clear thermodynamic driving force for the formation of a stable Sm-*V*_O_ complex at the 2NN separation. This energetic preference can be rationalized by considering the competition between electrostatic interaction and lattice relaxation. The substitution of Ce^4+^ by Sm^3+^ introduces a local negative charge, while the oxygen vacancy behaves effectively as a positively charged defect. Their Coulomb attraction favors defect association. However, in the 1NN configuration, strong local lattice distortion and steric repulsion around the vacancy site partially offset this electrostatic stabilization, making it less favorable than the 2NN configuration. At larger separations (3NN and 4NN), the interaction becomes negligible due to reduced electrostatic coupling and diminished lattice interaction.

**Figure 2:**
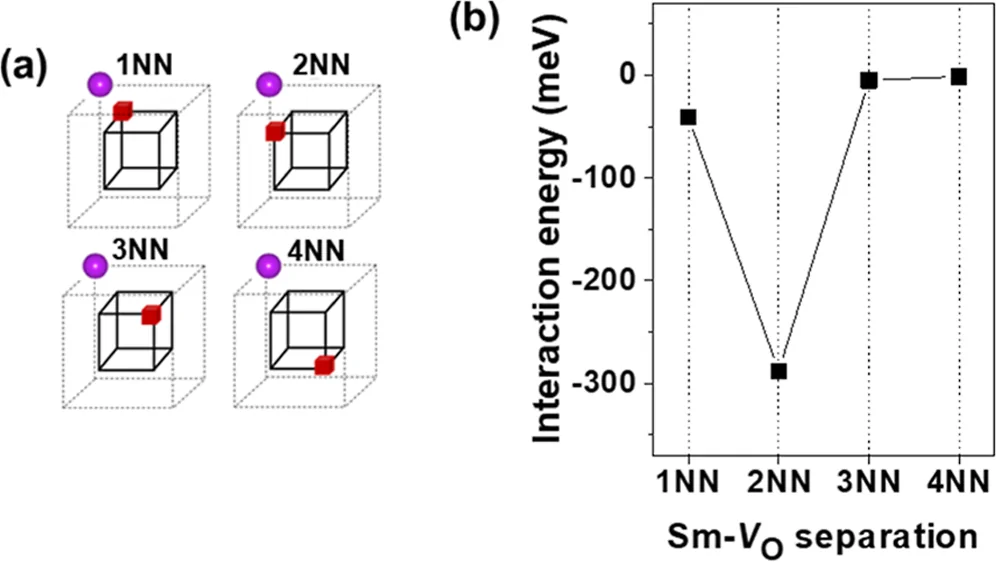
(Color online) (a) Schematic configurations of Sm-*V*_O_ distributions in CeO_2_, where *V*_O_ is located at the first (1NN), second (2NN), third (3NN) and fourth (4NN) nearest-neighbor positions relative to the Sm dopant. The thin-line and bold-line cubes denote the Ce and O sublattices, respectively. For clarity, Ce and O atoms are omitted, and only Sm and oxygen vacancy are highlighted by the violet sphere and red cube, respectively. (b) Calculated Sm-*V*_O_ interaction energies for four configurations.

It is well-known that an oxygen vacancy in CeO_2_ can exist in three charge states: neutral (*V*_*O*_^0^), singly charged (*V*_*O*_^+^), and doubly charged (*V*_*O*_^2+^). Accordingly, the Sm-*V*_O_ complex was examined under these three charge states, denoted as ${CeO_{2}:Sm+V}_{O}^{0}$ , ${CeO_{2}:Sm+V}_{O}^{+}$ , ${CeO_{2}:Sm+V}_{O}^{2+}$ , respectively. For the neutral vacancy ( ${CeO_{2}:Sm+V}_{O}^{0}$ ), two defect states emerge within the band gap, as shown in Figure 3a. The PDOS analysis (in-set of Figure 3a) reveals that these in-gap states are dominated by Ce 4f orbitals. Further charge-density analysis (Figure 4a) demonstrates that the corresponding electronic states are strongly localized on two Ce ions adjacent to the oxygen vacancy. Therefore, these gap levels mainly originates from the localized 4f states of two Ce ions surrounding *V*_O_. Quantitative integration shows that each of these Ce ions gains approximately 0.88e relative to the corresponding ion in CeO_2_:Sm, confirming their reduction from Ce^4+^ to Ce^3+^. This behavior indicates that the two excess electrons left behind by oxygen removal are not trapped directly at the vacancy site, but instead localize on nearby Ce ions. For the singly charged vacancy ( ${CeO_{2}:Sm+V}_{O}^{+}$ ), one of the two in-gap states disappears, as shown in Figure 3b, reflecting the removal of one excess electron from the neutral vacancy configuration. The remaining in-gap state is predominantly derived from the 4f orbital of a single Ce ion adjacent to *V*_O_, as confirmed by the PDOS analysis (in-set of Figure 3b) and the charge-density distribution in Figure 4b. Consequently, only one localized Ce^3+^ center persists in the system, which is consistent with the loss of one electron relative to the neutral vacancy case. In contrast, for the doubly charged vacancy ( ${CeO_{2}:Sm+V}_{O}^{2+}$ ), no defect states are observed within the band gap (Figure 3c), indicating that both excess electrons have been removed and all Ce ions remain in the Ce^4+^ state. These results highlight a fundamental feature of CeO_2_: excess electrons associated with oxygen vacancies preferentially localize on nearby Ce ions, forming small polarons (Ce^3+^ states), rather than residing at the vacancy site. This polaronic localization plays a crucial role in determining both the electronic structure and magnetic behavior.

**Figure 3:**
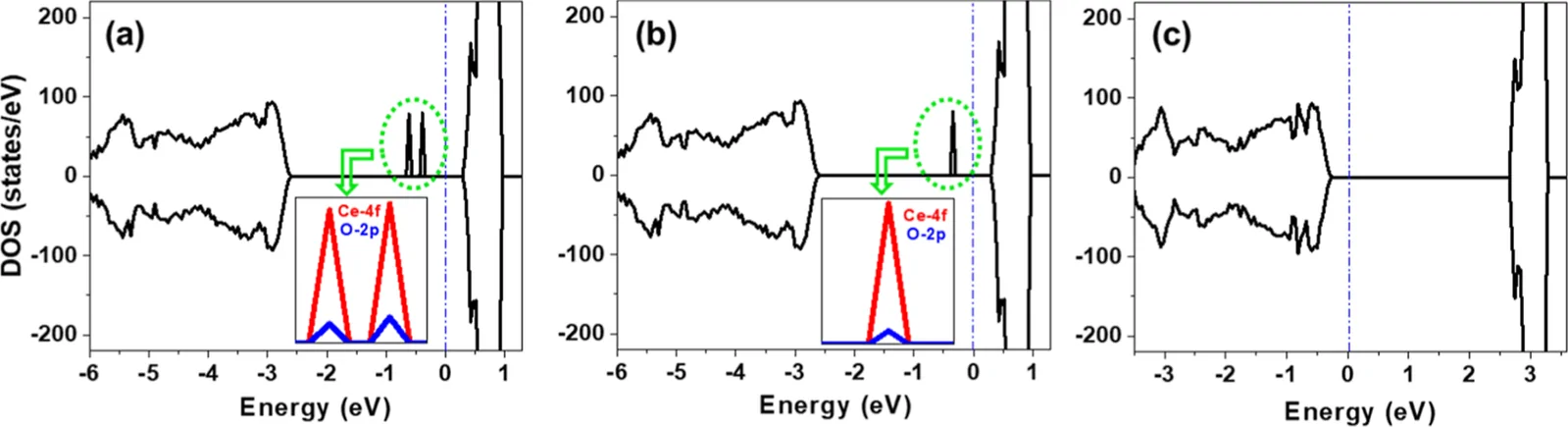
(Color online) DOSs of 2 × 2 × 2 CeO_2_ supercells doped with a Sm-*V*_O_ complex with (a) neutral, (b) singly charged, and (c) doubly charged oxygen vacancies. The dotted-dashed line at energy zero represents the Fermi level. The insets in panels (a) and (b) present the PDOS analyses of the localized in-gap states. The inset in (a) shows the PDOS of the 4f orbitals (red) for the two Ce ions nearest to *V*_O_, as well as the PDOS of the 2p orbitals (blue) for the bridging O ion linking the two Ce ions. The inset in panel (b) displays the corresponding PDOS (red) of the 4f orbitals associated with the Ce ion adjacent to *V*_O_, along with the PDOS of O 2p states (blue).

**Figure 4:**
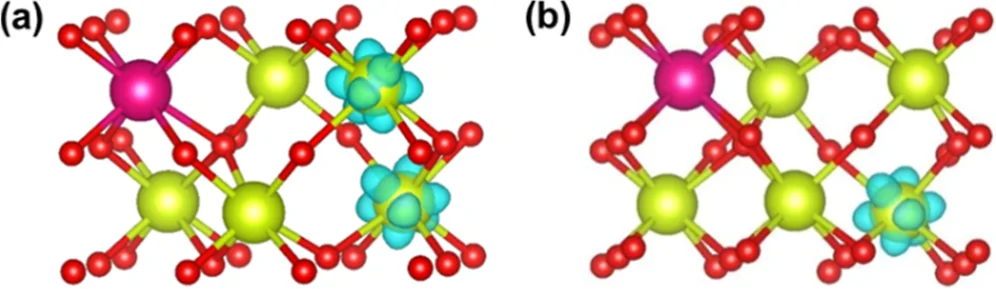
(Color online) Charge-density analyses for the gap states in the systems doped with a Sm-*V*_O_ complex with (a) neutral and (b) singly charged oxygen vacancies. Light blue isosurfaces represent the charge density of 0.1 e/Å^3^.

We next analyze the magnetic properties associated with these defect configurations. Since the DOS of ${CeO_{2}:Sm+V}_{O}^{2+}$ remains spin-symmetric (indicating a nonmagnetic ground state), the discussion focuses on the neutral and singly charged vacancy cases. For ${CeO_{2}:Sm+V}_{O}^{0}$ , the presence of two neighboring Ce^3+^ ions introduces two localized magnetic monments. To identify the magnetic ground state associated with these two localized moments, two spin configurations were considered: a ferromagnetic (FM, ↑↑) alignment and an antiferromagnetic (AFM, ↑↓) alignment. The calculated total energies show that the FM configuration is energetically favored over the AFM state by 38 meV, indicating a weak but finite tendency toward ferromagnetic coupling between the two Ce^3+^ ions. This energy preference is consistent with the spin-resolved DOS results (Figure 3a), where the defect-induced Ce 4f states are predominantly polarized within the same spin channel. This magnetic interaction can be understood in terms of a Ce 4f–O 2p–Ce 4f superexchange mechanism. As shown in Figures 4a and 5, the lobes of the Ce 4f orbitals are oriented toward different 2p orbitals of a shared O ion (which are commonly nearest neighboring to both Ce^3+^ ions), forming an indirect Ce 4f–O 2p–Ce 4f exchange pathway. This interpretation is further supported by the evident hybridization between the Ce 4f orbitals and the O 2p states in the in-set of Figure 3a, where the in-gap states exhibit mixed Ce 4f/O 2p character. The corresponding Ce–O–Ce bond angle (∼108°) is considerably closer to 90° than to 180°, which favors ferromagnetic superexchange according to Goodenough–Kanamori–Anderson rules.^40–42^ In such a near-90° geometry, the two Ce 4f electrons interact predominantly through different 2p orbitals of the same O ion. Owing to Hund’s intra-atomic exchange on the O ion, electrons occupying different 2p orbitals tend to align their spins parallel, which in turn promotes parallel spin alignment between the neighboring Ce^3+^ ions, as schematically shown in Figure 5b,c. It should be emphasized, however, that this interaction is relatively weak and highly localized, as reflected by the small ferromagnetic stabilization energy obtained above. This is further supported by the spin-density distribution (Figure 5a), which shows that the magnetic moments are highly localized and limited in magnitude. Such weak and short-range interactions are unlikely to produce robust ferromagnetic ordering. Therefore, these results suggest that magnetism mediated solely by the neutral oxygen vacancy is insufficient to explain the strong room-temperature ferromagnetism observed experimentally in Sm-doped CeO_2_.^13,14,43^

**Figure 5:**
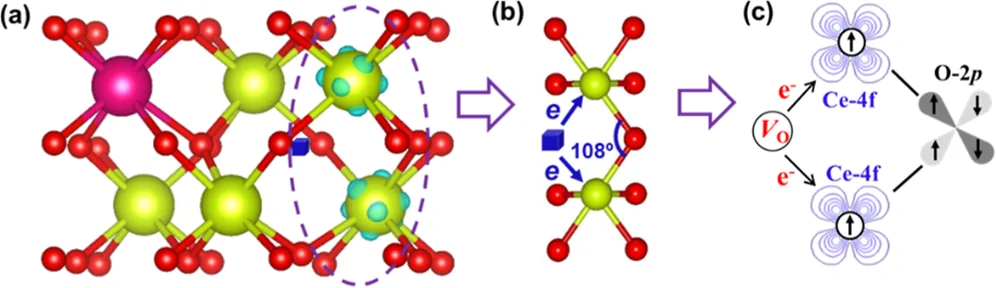
(Color on line) (a) Spin density of the system doped with a Sm-*V*_O_ complex with a neutral oxygen vacancy. Oxygen vacancy is represented by a blue cube. Light blue isosurfaces appearing on two Ce^3+^ ions represent the charge density of 0.1 e/Å^3^. (b) and (c) Schematic for the superexchange interaction between two Ce^3+^ ions.

Upon introducing a singly charged oxygen vacancy (*V*_*O*_^+^) in the CeO_2_:Sm system ( ${CeO_{2}:Sm+V}_{O}^{+}$ ), only a single spin-polarized state appears within the band gap, as shown in Figure 3b. This state is clearly associated with a localized Ce^3+^ center. The corresponding spin-up state gives rise to a magnetic moment of ∼0.86 μ_B_, in good agreement with the spin-density distribution (Figure 6). The spin density is highly localized on a Ce ion adjacent to the vacancy and exhibits a characteristic 4f orbital shape, while negligible charge accumulation is observed at the vacancy site itself. This unambiguously indicates that the excess electron does not form a vacancy-centered F^+^ center, but instead localizes on a nearby Ce ion, forming a small polaron (Ce^3+^). This behavior reflects the strong localization tendency of Ce 4f electrons and provides direct evidence for the formation of bound magnetic polarons.^44–46^ Therefore, the experimentally observed ferromagnetism in Sm-doped CeO_2_ is more plausibly attributed to BMP-mediated exchange interactions rather than the conventional FCE mechanism, although the latter may still operate under specific conditions where electrons remain trapped at vacancy sites.

**Figure 6:**
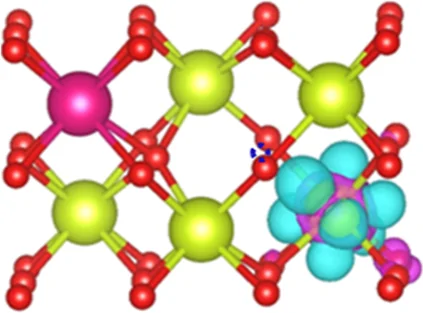
(Color on line) Spin density of the system doped with a Sm-*V*_O_ complex with a singly charged oxygen vacancy. Light blue and magenta isosurfaces represent the charge density of positive and negative 0.1 e/Å^3^, respectively. Oxygen vacancy is represented by a blue dot circle.

From an energetic perspective, the stability of oxygen vacancies in different charge states is evaluated using the standard defect formation energy formalism^47^

2 $$\Delta E_{f}\left(V_{O}^{q}\right)=E\left(CeO_{2}:{Sm+V}_{O}^{q}\right)-E\left(CeO_{2}:Sm\right)+\mu_{O}+q\left(E_{F}+E_{VBM}\right)$$

where *E*(CeO_2_:*Sm* + *V*_*O*_^*q*^) and *E*(*CeO*_2_:*Sm*) are the total energies of the Sm-doped CeO_2_ supercells with and without an oxygen vacancy in charge state *q*, respectively. *E*_F_ is the Fermi level referenced to the valence band maximum (*E*_VBM_), and μ_*O*_ is oxygen chemical potential. The latter is expressed as μ_*O*_ = *E*(*O*)+Δμ_*O*_, where *E*(*O*) is the total energy of an isolated oxygen atom, and the extraneous chemical potential Δμ_*O*_ is constrained by the thermodynamic equilibrium conditions: Δμ_Ce_+2Δμ_*O*_ = *H*_*f*_(CeO_2_), together with the condition Δμ_*O*_ ≤ 0 to avoid O_2_ precipitation. Here, *H*_*f*_(CeO_2_) denotes the formation enthalpy of CeO_2_. Under O-rich conditions, Δμ_*O*_ = 0 eV (corresponding to an oxygen-saturated environment), whereas under O-poor (reducing) conditions—where CeO_2_ is in equilibrium with Ce_2_O_3_—the chemical potentials are further constrained by 2Δμ_Ce_+3Δμ_*O*_ = *H*_*f*_(Ce_2_O_3_), which leads to a lower value of Δμ_*O*_. The calculated formation energies of *V*_O_ in charge states 0, +1 and +2 for pure and Sm-doped CeO_2_ under the O-poor and O-rich conditions are presented in Figure 7a,b, revealing two key trends. First, the chemical environment plays a decisive role in vacancy formation: O-poor conditions strongly favor the vacancy formation, whereas O-rich conditions significantly suppress it. Second, irrespective of the chemical environment (O-poor or O-rich conditions), the introduction of Sm dopants significantly lowers the formation energies of *V*_O_ in all charge states, consistent with the role of Sm^3+^ as an efficient promoter of oxygen vacancy formation.^13,14^ More importantly, in the presence of Sm doping (red lines in Figure 7), the 1+ charge state becomes thermodynamically stable over a wide Fermi-level range. This stabilization originates from the aliovalent substitution of Ce^4+^ by Sm^3+^, which introduces an effective negative charge into the lattice and shifts the charge balance toward partially compensated vacancy states. As a result, the singly charged vacancy is energetically favored compared to the neutral and doubly charged states under a broad range of conditions. The *V*_*O*_^+^ state is of particular importance because it hosts a single unpaired electron (spin 1/2), which localizes on a neighboring Ce ion, forming a Ce^3+^ small polaron. This localized magnetic entity constitutes a bound magnetic polaron, which serves as the fundamental magnetic building block in the system. Compared to the neutral vacancy (where two electrons remain magnetically weakly coupled), the *V*_*O*_^+^ state provides a more effective and spatially flexible magnetic center.

**Figure 7:**
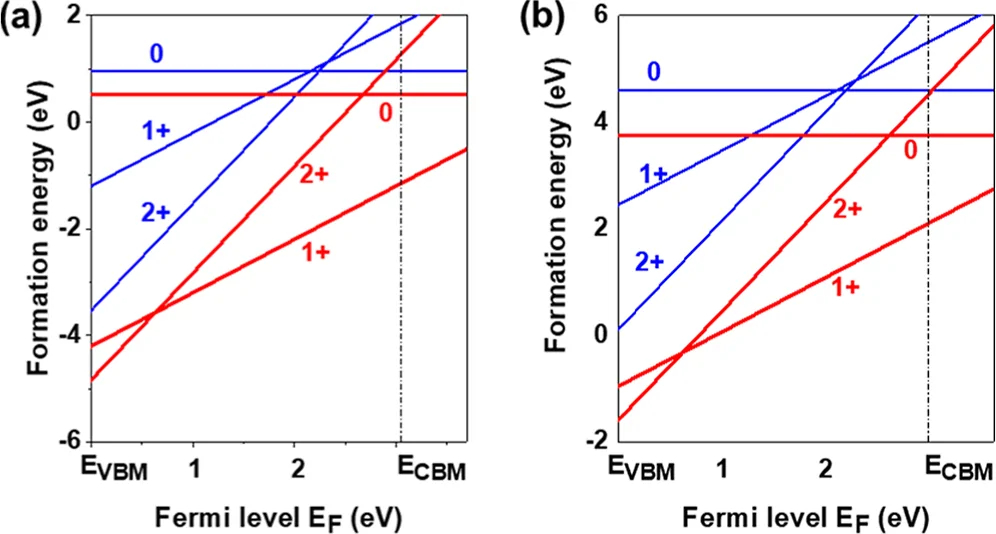
(Color online) The calculated formation energies of oxygen vacancies in different charge states (0, 1+, and 2+) as a function of the Fermi level in a 2 × 2 × 2 CeO_2_ supercell. Two chemical environments are considered: (a) O-poor and (b) O-rich conditions. The results for the Sm-doped system with a Sm–*V*_O_ complex are shown as red bold lines, while those for pure CeO_2_ are plotted as blue lines for comparison.

The spatial extent of this BMP is governed by the polaron radius (*r*_*p*_), which can be estimated as^48^

3 $$r_{p}=\frac{\hbar }{\sqrt{2m^{*}E_{p}}}$$

where ℏ is the reduced Planck constant, m* is the effective electron mass, *E*_p_ is the polaron binding energy, respectively. Using the hybrid-functional-derived value m* = 2.1 *m*_0_ and *E*_p_ = 0.28 eV (evaluated following Kokott et al.^49^), the polaron radius is estimated to be ∼2.6 Å, indicative of a strongly localized small polaron rather than a large, delocalized one. This suggests that, to achieve the spatial overlap and percolation between neighboring polarons and cause ferromagnetism to emerge, the BMP concentration needs to exceed a critical percolation threshold (η_*c*_ ≈ 0.29) according to the continuum percolation theory.^50,51^ The critical BMP concentration (*n*_*BMP*_) can be evaluated using the condition

4 $$n_{BMP}\cdot \frac{4}{3}\pi r_{p}^{3}\approx \eta_{c}$$

where η_*c*_ ≈ 0.29 is the critical volume fraction for three-dimensional percolation. The critical *n*_*BMP*_ is calculated to be 4.0 × 10^21^ cm^–3^, indicating that a sufficient high defect density is required to achieve percolation. For the sake of comparison, we also calculate the *V*_O_ concentration represented by our supercell model. For the defect-containing models, one oxygen vacancy is introduced into the same supercell (Ce_32_O_63_), corresponding to an oxygen-vacancy concentration of approximately 1.56% of the oxygen sublattice (1/64), or equivalently ∼2.9 × 10^20^ cm^–3^ based on the calculated lattice parameter. This value is approximately 1 order of magnitude lower than the critical BMP concentration, indicating that the current model does not correspond to a percolated BMP network. Rather, it represents an isolated Sm–*V*_O_ complex that allows us to identify the microscopic origin of magnetic-polaron formation and to establish the defect chemistry responsible for stabilizing the *V*_*O*_^+^ charge state. The formation of long-range ferromagnetic ordering through BMP overlap therefore requires substantially higher concentrations of Sm dopants and oxygen vacancies.

It should be pointed out that this dilute Sm concentration (3.13%) is appropriate for revealing the intrinsic Sm–*V*_O_ interaction while minimizing direct dopant–dopant coupling. Although the present model does not quantitatively describe heavily doped systems, it suggests that increasing the Sm concentration would be expected to increase the density of oxygen vacancies and the associated BMPs, provided that phase stability and defect clustering do not become dominant. This interpretation is qualitatively consistent with experimental reports^16,24^ that the strongest room-temperature ferromagnetism is often observed at Sm concentrations of approximately 15–20%. In addition to dopant concentration, the chemical environment plays a crucial role. The low formation energies of *V*_*O*_^+^ under oxygen-poor conditions (Figure 7a) suggest that a high density of singly charged vacancies—and thus BMPs—can be readily achieved in reducing environments. This provides a natural explanation for the robust ferromagnetism observed experimentally in samples synthesized or annealed under oxygen-deficient conditions.^13,43^

## 4 Conclusions

In summary, a hybrid functional study has been conducted to elucidate the role of Sm doping and oxygen vacancies in governing the electronic structure and magnetism of Sm-doped CeO_2_. Results are consistent with a mechanism in which the experimentally observed ferromagnetism in Sm-doped CeO_2_ is mediated by the synergistic interplay between dopants and oxygen-vacancy charge states, in which Sm acts primarily as a vacancy stabilizer rather than a direct magnetic contributor. The defect–dopant interaction identified in the system with a dilute Sm concentration provides a microscopic framework that is qualitatively consistent with experimentally observed concentration-dependent ferromagnetism. Although quantitative prediction of concentration-dependent magnetic properties will require future large-supercell calculations including explicit dopant–dopant interactions, the proposed BMP-mediated interpretation therefore offers a clear defect-engineering framework: ferromagnetism in Sm-doped CeO_2_ can be optimized by (i) increasing Sm doping concentration to promote vacancy formation and BMP density, (ii) employing reducing atmospheres during synthesis to lower *V*_*O*_^+^ formation energy. These insights extend beyond Sm-doped CeO_2_ and provide general design principles for developing ferromagnetic oxides through controlled interplay between aliovalent dopants and oxygen vacancies, with potential applications in spintronics and magnetic oxide electronics.
